# 高降解效率分子印迹光催化剂的制备及其选择性降解性能

**DOI:** 10.3724/SP.J.1123.2025.06001

**Published:** 2026-01-08

**Authors:** Junjie ZHANG, Yafei SONG, Yan LIU, Xuemeng TIAN, Ruixia GAO

**Affiliations:** 1. 西安交通大学化学学院，陕西 西安 710049; 1. School of Chemistry，Xi’an Jiaotong University，Xi’an 710049，China; 2. 西安交通大学第二附属医院，陕西 西安 710114; 2. The Second Affiliated Hospital，Xi’an Jiaotong University，Xi’an 710114，China

**Keywords:** 分子印迹, 选择性去除, 聚吡咯, 溴氧化铋, molecular imprinting, selective removal, polypyrrole, bismuth oxobromide

## Abstract

分子印迹光催化剂（MIPC）兼具分子识别选择性和光催化降解性能，在复杂环境中痕量污染物的选择性及深度去除方面展现出广阔的应用前景。然而，传统印迹层覆盖于光催化剂表面可能引发光屏蔽效应，进而导致MIPC光降解效率降低。针对这一问题，本工作提出异质界面原位印迹策略，通过将印迹空穴直接构筑于光催化剂/复合材料的界面处，有效规避表面覆盖层的不利影响，并促进光生载流子迁移，进而实现高选择性识别与高效光降解去除性能的协同提升。以偶氮类染料酸性橙为模板分子，BiOBr-Cu/聚吡咯复合材料为异质结体系，通过表面印迹技术，构建具有高降解效率的分子印迹光催化剂。采用扫描电镜、X-射线衍射图、红外光谱、X射线光电子能谱、紫外可见漫反射光谱、光致发光光谱等表征手段证明该材料成功制备，并进一步探究其吸附性能、降解性能、选择性降解性能及机理。所制备的材料不仅具有优异的可见光响应能力、快速的光生电子空穴分离效率，还具有高降解效率（比其他材料高出2.04~5.79倍）、良好的吸附容量（40.9 μmol/g）、快速的吸附速率（44.8 mg/（g·min））、良好的重复利用性（5个循环后，仍然能够达到初次降解率的90.7%）和优异的选择性（印迹因子达2.96，选择性降解参数大于1.79）。本工作为高降解效率分子印迹光催化材料的设计提供了新思路。

## Abstract (en)

Molecularly imprinted photocatalysts （MIPCs）， which integrate specific molecular recognition with photocatalytic degradation capabilities， hold great promise for the selective and efficient removal of trace pollutants from complex environmental matrices. However， conventional surface-imprinting layers coated on photocatalysts often cause light-shielding effects， thereby reducing the photocatalytic efficiency of MIPCs. To overcome this limitation， the present study proposes a heterojunction-based interfacial in-situ imprinting strategy. In this approach， molecular imprinting cavities are directly constructed at the interface of composite photocatalysts. This design not only avoids the negative impact of surface shielding but also facilitates interfacial charge transfer， thus achieving a synergistic enhancement of both molecular recognition selectivity and photocatalytic efficiency. Based on this concept， a highly efficient molecularly imprinted photocatalyst， BiOBr-Cu/ppyr-MIPs， was successfully fabricated. The synthesis utilized surface molecular imprinting technology with the organic pollutant acid orange （AO） as the template molecule， and BiOBr-Cu/polypyrrole （ppyr） composite as the heterojunction matrix. Polypyrrole， introduced as a conductive polymer， served as the imprinting layer that promotes charge migration. The synthesis conditions， including monomer amount and polymerization time， were systematically optimized to maximize imprinting efficiency and photocatalytic performance. The obtained BiOBr-Cu/ppyr-MIPs were comprehensively characterized. Scanning electron microscopy （SEM） revealed a well-defined morphology with uniformly distributed imprinted layers. X-ray diffraction （XRD） analysis confirmed that the introduction of the polypyrrole-based imprinting layer did not significantly alter the crystal structure of the BiOBr-Cu composite. Fourier-transform infrared spectroscopy （FTIR） showed characteristic peaks attributable to the pyrrole ring， such as C-H in-plane bending， C-H stretching， and C=N stretching vibrations， indicating successful incorporation of the polypyrrole framework. X-ray photoelectron spectroscopy （XPS） further confirmed the formation of the imprinted layer， with increased proportions of C-N and O=C bonding components observed in the C 1*s* and O 1*s* spectra， respectively. Optical and photoelectronic properties were also evaluated. UV-vis diffuse reflectance spectroscopy （DRS） revealed a significant red-shift and broader light absorption in the visible range， attributed to the presence of the polypyrrole layer. Photoluminescence （PL） spectroscopy demonstrated a marked decrease in emission intensity， indicating that the recombination of photogenerated electron-hole pairs was effectively suppressed， which correlates with enhanced charge separation. Adsorption experiments indicated that BiOBr-Cu/ppyr-MIPs exhibited rapid adsorption kinetics， reaching equilibrium within 30 min and fitting a pseudo-second-order kinetic model. The maximum adsorption capacity was determined to be 40.9 μmol/g， consistent with the Freundlich isotherm model， suggesting a heterogeneous adsorption process with multilayer adsorption behavior. Photocatalytic degradation studies， optimized by adjusting catalyst dosage， initial pollutant concentration， and solution pH， showed a 2.04-5.79-fold enhancement in degradation efficiency compared to reference materials. The composite also exhibited excellent reusability， maintaining 90.7% of its initial performance after five consecutive cycles. Importantly， the material demonstrated strong molecular recognition capability， with an imprinting factor （IF） of 2.96 and a selectivity coefficient （*K*_selectivity_） exceeding 1.79， indicating effective discrimination between the template molecule and structural analogs. Mechanistic investigations revealed that the interfacial polypyrrole imprinting layer not only contributed to selective adsorption but also facilitated targeted degradation pathways， thus achieving integrated selectivity and catalytic activity. In summary， this work introduces a novel interfacial in-situ imprinting strategy that overcomes key limitations of conventional MIPCs. The proposed design offers a generalizable approach for developing molecularly imprinted photocatalysts with both superior degradation efficiency and selectivity.

随着工业化与城市化的加速推进，环境水体中有害污染物的持续积累问题日益严峻^［1，2］^。尽管这些污染物在环境水体中的浓度通常较低，但其显著的毒性对生态系统和人类健康构成了严重威胁^［3］^。因此，开发高效、选择性地去除此类污染物的新方法显得尤为关键。分子印迹聚合物（molecularly imprinted polymers，MIPs）因具有特异性识别位点，在痕量污染物的富集和分离方面展现出良好应用前景^［4］^。然而，传统MIPs仅能实现污染物的相转移，难以实现其彻底降解，若后处理不当或污染物泄漏，可能引发二次污染风险^［5］^。因此，构建兼具高选择性识别与高效去除能力的多功能MIPs材料具有重要意义。

分子印迹光催化剂（molecularly imprinted photocatalyst，MIPC）是将光催化技术引入MIPs体系的新型功能材料，为实现污染物选择性识别与深度去除的协同治理提供了新策略。自Shen等^［6］^首次在TiO_2_表面构建印迹层以来，基于半导体材料的MIPC迅速发展。TiO_2_、ZnO等宽禁带半导体材料因具有优良的稳定性与催化性能，被广泛用作MIPC的光催化载体^［7-9］^。然而，纯相宽禁带半导体仍面临太阳光利用率低、光生载流子复合速率高等问题，限制其降解效率。本课题组的前期研究发现^［10］^，铜掺杂的BiOBr光催化剂具有优异的可见光响应性能和高效的载流子分离能力，具备成为理想MIPC光催化载体的潜力。

此外，在MIPC结构中，印迹层的构建是实现高选择性的关键，而功能单体则是印迹层形成的核心因素。目前常用的功能单体如丙烯酸、甲基丙烯酸、丙烯酰胺等^［11-14］^虽可赋予材料良好的选择性识别能力，但其在光催化剂表面形成的聚合层可能阻碍入射光的吸收和/或光生载流子迁移等不利影响，进而导致MIPC降解效率降低。构建异质结结构被认为是提升光催化性能的有效途径，其能促进光生载流子的有效分离^［15］^。受此启发，本研究提出“异质界面原位印迹”策略，将印迹空穴直接构筑于光催化剂/复合材料的界面处。该策略有效规避了表面覆盖层的不利影响，并利用印迹层-光催化载体形成的异质结构促进光生载流子迁移，从而实现高选择性识别与高效光降解去除性能的协同提升。

基于此，本工作以典型的偶氮类染料酸性橙为模板分子，以BiOBr-Cu/聚吡咯复合材料为异质结体系，采用表面印迹技术，构建了高降解效率分子印迹光催化剂，即分子印迹吡咯/溴氧化铋复合材料（BiOBr-Cu/ppyr-MIPs）。聚吡咯层的引入不仅提供了对目标污染物的高选择性识别位点，而且能够作为光生空穴迁移通道，有效抑制电子-空穴对的复合，从而增强整体光催化活性。本研究对材料的结构、吸附、光催化性能及选择性进行了系统优化与表征，为开发兼具高选择性和高效去除能力的分子印迹材料提供新思路。

## 1 实验部分

### 1.1 仪器、试剂与材料

GeminiSEM 500场发射扫描电子显微镜（SEM，德国蔡司公司），D8 ADVANCE X射线衍射仪（XRD，德国Bruker公司），VERTEX70傅里叶变换红外光谱仪（FTIR，德国Bruker公司），ESCALAB Xi+X射线光电子能谱仪（XPS，美国Thermo Fisher公司），PE Lambda950紫外-可见-近红外分光光度计（UV-vis DRS，美国PerkinElmer公司），FLS980瞬态稳态荧光光谱仪（PL，英国爱丁堡仪器公司），LCMS-2020液相色谱-质谱联用仪（LC-MS，日本岛津公司）。

三水合硝酸铜（Cu（NO_3_）_2_·3H_2_O，≥99%）、五水合硝酸铋（Bi（NO_3_）_3_·5H_2_O，分析纯）、溴化钾（KBr，分析纯）、聚乙烯吡咯烷酮（PVP，平均相对分子质量58 000）、偶氮二异丁腈（AIBN，≥99%）、三羟甲基丙烷三甲基丙烯酸酯（TRIM，≥90%）、氢氧化钠（NaOH，分析纯）购自上海阿拉丁有限公司。吡咯（pyr，99%）购自上海麦克林有限公司。酸性橙（AO，85%）、阿落拉红（AR，98%）、苋菜红（RA，85%）、橙黄G（OG，≥96%）、三氯甲烷（CHCl_3_，分析纯）、三乙醇胺（TEOA，>99%）、异丙醇（IPA，99.9%）、L-抗坏血酸（LAA，99.9%）、盐酸（HCl，分析纯）购自西安化工有限公司。无水乙醇（分析纯）、乙二醇（分析纯）购自天津市富宇精细化工有限公司。实验全过程使用超纯水（18.25 MΩ·cm）。

### 1.2 材料制备

将240.0 mg Bi（NO_3_）_3_·5H_2_O、122.0 mg KBr、1 000.0 mg PVP、3.7 mg Cu（NO_3_）_2_·3H_2_O置于70 mL乙二醇中，分散均匀后转移至聚四氟乙烯反应釜中，在140 ℃下反应10 h。反应结束后，用超纯水和无水乙醇交替洗涤产物，于60 ℃下真空干燥，得到浅绿色沉淀BiOBr-Cu^［16］^。

准确称取100.0 mg BiOBr-Cu、15.0 mg AO于三口烧瓶中，加入30 mL超纯水，磁力搅拌30 min。然后，将功能单体6 mmol pyr、交联剂0.3 mL TRIM和引发剂0.3 mmol AIBN超声分散于1 mL三氯甲烷中，逐滴加入上述三口烧瓶中，继续搅拌30 min。最后，向三口烧瓶中持续通入氮气，于75 ℃下加热反应24 h，用超纯水洗涤产物，于60 ℃下真空干燥。将干燥后的产物置于双层夹套烧杯中，加入50 mL超纯水分散，光照下降解去除模板分子。离心分离得到BiOBr-Cu/ppyr-MIPs。

作为对比，在不加入模板分子AO的条件下制备非印迹吡咯/溴氧化铋复合材料（BiOBr-Cu/ppyr-NIPs），其他步骤同上。

### 1.3 材料的吸附性能考察

动力学吸附实验：取10.0 mg BiOBr-Cu/ppyr-MIPs或BiOBr-Cu/ppyr-NIPs，加入到30.0 mL质量浓度为20.0 μg/mL的AO溶液中，在室温下遮光、振荡吸附后，离心分离上清液并测定AO的吸光度。

热力学吸附实验：取10.0 mg BiOBr-Cu/ppyr-MIPs或BiOBr-Cu/ppyr-NIPs，加入到不同质量浓度的30.0 mL AO溶液中，在室温下遮光、振荡吸附30 min后，离心分离上清液并测定AO的吸光度。

根据式（1）和式（2）计算吸附量和印迹因子（imprinting factor， IF）。


*Q*=(*C*_0_-*C*_t_)/(*V*·*W*) (1)

IF=*Q*_MIP_/*Q*_NIP_ (2)

其中，*Q*（mg/g）为材料对目标物的吸附量；*C*_0_和*C_t_* （μg/mL）分别为目标物初始浓度和吸附时间为*t*时的质量浓度；*V*（mL）为溶液的体积；*W*（mg）代表材料质量；*Q*_MIP_和*Q*_NIP_（mg/g）分别为印迹材料BiOBr-Cu/ppyr-MIPs和非印迹材料BiOBr-Cu/ppyr-NIPs对目标物的吸附量。

### 1.4 材料的降解性能考察

取BiOBr-Cu/ppyr-MIPs于双层夹套烧杯中，加入30 mL AO溶液（10.0、20.0、30.0、40.0 μg/mL），放置于磁力搅拌器上，用锡纸避光，在黑暗环境下反应30 min至吸附-脱附平衡。打开冷却循环水，开启光源，每隔30 min取少量悬浮液，离心后使用紫外-可见分光光度计测量吸光度，计算残留目标物浓度。根据式（3）计算降解率，根据准一级动力学方程式（4）对降解过程进行分析，根据式（5）计算特异性识别系数（*k*_recognition_）。通过式（6）计算光催化材料的转换频率（turnover frequency， TOF， min^-1^），对光降解性能相关的数据归一化分析。


*η*=(*C*_0_–*C*_dt_)/*C*_0_ (3)

ln *C*_dt_/*C*_1_=*kt* (4)

*k*_recognition_=*η*_MIPs_/*η*_NIPs_ (5)

TOF=*W*_p_/*W*_c_
*t* (6)

其中，*η*为降解率；*C*_dt_（μg/mL）为降解*t*时间后目标物的质量浓度；*C*_1_为吸附平衡后目标物浓度；*k*（min^-1^）为一级动力学速率常数；*η*_MIPs_和*η*_NIPs_分别为印迹材料和非印迹对目标分子的降解率；*W*_c_（mg）和*t*（min）分别为初始材料用量和光降解过程持续时间；*W*_p_（mg）为光照时间为*t*时降解去除的污染物分子的质量。

### 1.5 选择性实验

选取结构类似物AR、RA、OG作为竞争物，评估BiOBr-Cu/ppyr-MIPs对AO的降解选择性。将15.0 mg BiOBr-Cu/ppyr-MIPs或BiOBr-Cu/ppyr-NIPs加入到30.0 mL 20 μg/mL结构类似物溶液中，黑暗下吸附后进行光降解实验。根据式（7）、（8）、（9）计算选择性降解参数（*k*_selectivity_）以评估降解选择性。

对于印迹体系：


*k*_imprinted_=*η*_MIPs-T_/*η*_MIPs-C_ (7)

对于非印迹体系：


*k*_comparison_=*η*_NIPs-T_/*η*_NIPs-C_ (8)

对于降解选择性：


*k*_selectivity_
*=k*_imprinted_/*k*_comparison_ (9)

其中，T代表目标物，C代表竞争物。

### 1.6 选择性降解机理探究

#### 1.6.1 活性物种捕获实验

分别以LAA、IPA和TEOA为超氧阴离子自由基（·O_2_^-^）、羟基自由基（·OH）和空穴（h^+^）的捕获剂，探究光催化过程中的主要活性物种。将15.0 mg的BiOBr-Cu/ppyr-MIPs分散于30 mL 20.0 μg/mL的AO溶液中，分别加入1 mmol/L的LAA、IPA和TEOA，超声分散均匀，黑暗下吸附后进行光降解实验。

#### 1.6.2 降解中间产物分析

采用HPLC-MS对光催化降解过程中产生的中间产物进行测定。HPLC-MS测试条件如下：C18色谱柱（250 mm×4.6 mm，3 μm），流动相为甲醇-0.1%乙酸铵水溶液（90∶10，体积比），流速为0.4 mL/min，进样量为10 μL，柱温30 ℃。采用电喷雾电离模式进行质谱分析。

## 2 结果与讨论

### 2.1 BiOBr-Cu/ppyr-MIPs的制备

本工作将表面分子印迹技术引入BiOBr-Cu/聚吡咯复合材料制备过程中，通过固定模板法，制备得到高降解效率的分子印迹光催化剂BiOBr-Cu/ppyr-MIPs。BiOBr-Cu/ppyr-MIPs的制备过程及其对模板分子可能的识别机制如图1所示。首先，通过简单的溶剂热法，一步掺杂制备得到铜掺杂BiOBr花状微球（BiOBr-Cu）。然后，利用固定模板法，将模板分子AO固定在BiOBr-Cu表面，形成BiOBr-Cu/AO复合物。随后，加入功能单体pyr、交联剂TRIM和引发剂AIBN形成聚吡咯层，通过多重氢键和*π-π*相互作用将AO进一步固定在BiOBr-Cu表面。聚吡咯印迹层不仅具有丰富的官能团，有助于形成大量与模板分子在形状、大小及功能基团上相匹配的印迹位点，同时具有良好的可见光吸收特性和优异的空穴传输能力，从而协同光催化载体提高光利用率。最后，通过光洗脱去除模板分子，得到BiOBr-Cu/ppyr-MIPs。

**图 1:**
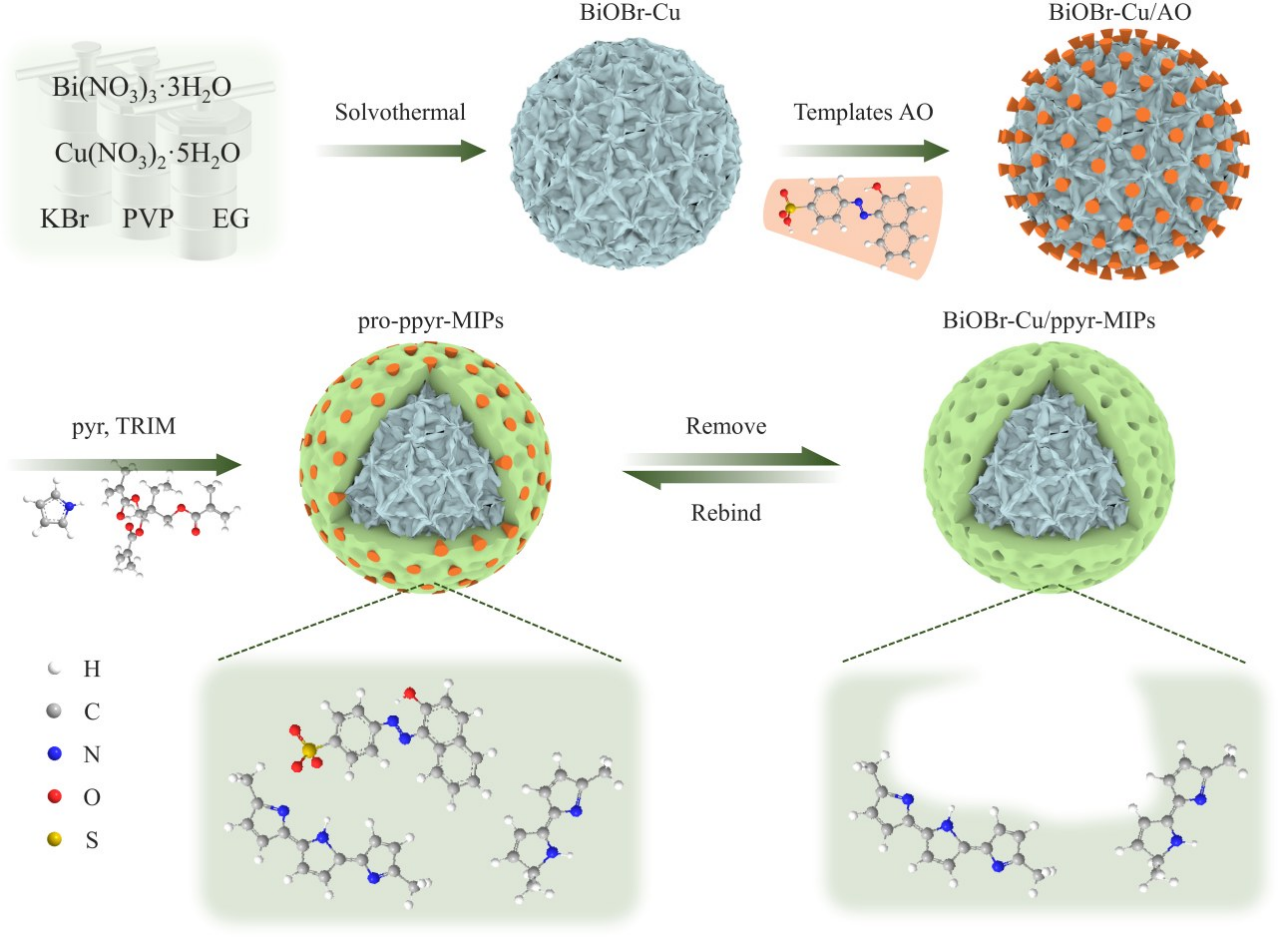
BiOBr-Cu/ppyr-MIPs的合成路线图

为了获得最优的选择性去除能力，对功能单体pyr的用量进行了优化，如图2所示。随着pyr用量的增加，BiOBr-Cu/ppyr-MIPs和BiOBr-Cu/ppyr-NIPs的降解率均呈现先增加后下降的趋势。这表明适量增加pyr用量可以增强材料的光催化活性。然而，当pyr用量过大时，光催化性能降低。这可能是过量的pyr覆盖在BiOBr-Cu载体表面，不仅阻碍其对可见光的吸收，而且会限制反应物的传质和扩散，从而导致光降解率降低^［17，18］^。相较于BiOBr-Cu/ppyr-NIPs，BiOBr-Cu/ppyr-MIPs始终保持较高的降解率，当pyr用量为6 mmol时，特异性识别系数*k*_recognition_最大，这可以归因于印迹位点的存在提供了特异性吸附和识别目标污染物的能力，从而有效提高了材料的光催化选择性和降解效率。在后续实验中，确定最佳的pyr用量为6 mmol。

**图2:**
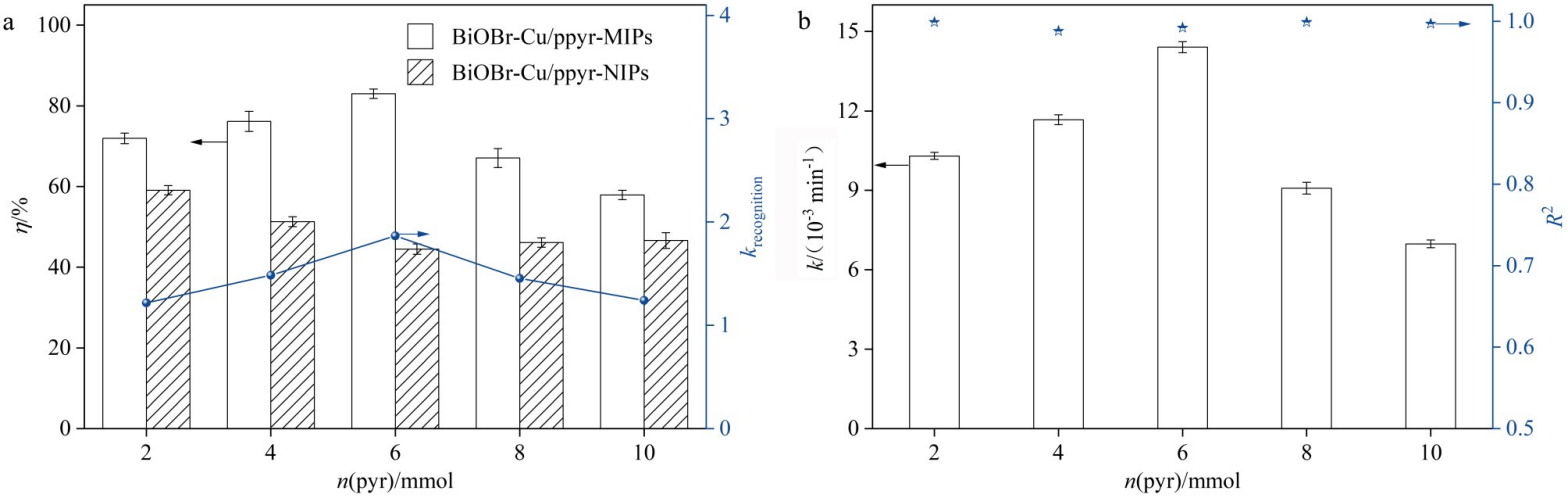
（a）功能单体pyr的用量对材料光降解性能的影响及（b）BiOBr-Cu/ppyr-MIPs动力学常数（*n*=3）

当pyr用量为6 mmol时，对聚合时间进行考察，如图3所示，聚合时间小于24 h时，降解率和*k*_recognition_均较低，表明形成的印迹位点数目有限，不能实现特异性识别目标污染物。随着聚合时间增加至24 h，BiOBr-Cu/ppyr-MIPs的降解率达到最大值，同时*k*_recognition_也显著增加。这可能是由于在印迹聚合物中，聚吡咯层形成了丰富的印迹位点，与目标污染物分子结合力增强，从而提高了材料的特异性识别能力和降解性能。继续延长聚合时间，BiOBr-Cu/ppyr-MIPs和BiOBr-Cu/ppyr-NIPs的降解率均下降，这可能是由于过多的聚合导致层厚增加，进而影响分子扩散和反应效率。因此在后续实验中，采用聚合时间为24 h的制备条件对BiOBr-Cu/ppyr-MIPs进行合成。

**图3:**
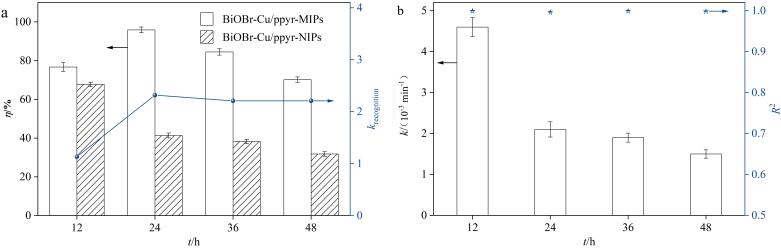
（a）聚合时间对光降解性能影响及（b）BiOBr-Cu/ppyr-MIPs动力学常数（*n*=3）

### 2.2 BiOBr-Cu/ppyr-MIPs表征

#### 2.2.1 SEM表征

通过SEM对BiOBr-Cu和BiOBr-Cu/ppyr-MIPs的形貌进行表征，如图4所示，可以明显看出，BiOBr-Cu和BiOBr-Cu/ppyr-MIPs的粒径较为均一，具有良好的分散性。BiOBr-Cu呈现出由纳米片自组装而成花状微球结构，直径约为2.45 μm。这种独特的三维结构已被报道能够通过多次光反射增强材料的光吸收^［19，20］^。在图4b中，BiOBr-Cu/ppyr-MIPs表面呈现松散的聚合物结构，且材料直径显著增加至4.54 μm，初步表明聚吡咯印迹层的成功包覆。

**图4:**
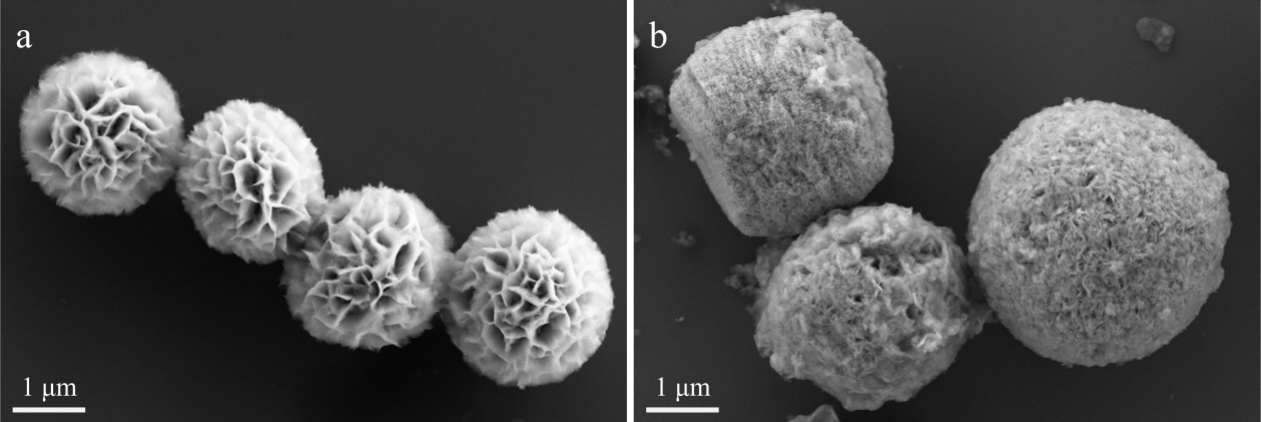
（a）BiOBr-Cu和（b）BiOBr-Cu/ppyr-MIPs的SEM图

#### 2.2.2 XRD表征

通过XRD对BiOBr-Cu和BiOBr-Cu/ppyr-MIPs的晶体结构进行表征，如图5a所示。在BiOBr-Cu样品中，XRD谱图中出现9个特征衍射峰，2*θ*值分别为25.2°、32.2°、39.4°、46.2°、53.4°、56.1°、57.1°、67.4°和76.7°。这些特征峰归属于晶面（1 0 1）、（1 1 0）、（1 1 2）、（2 0 0）、（2 1 1）、（1 1 4）、（2 1 2）、（2 2 0）和（3 1 0），与四方晶系BiOBr结构（JCPDS 09-0393）一致。此外，在2*θ*为29.5°处出现的衍射峰与单斜Bi_4_O_5_Br_2_的标准卡（JCPDS 37-0699）相符。这可能是由于在Cu掺杂过程中，部分Br被Cu取代，导致Bi与Br的比例增加。对于BiOBr-Cu/ppyr-MIPs样品，其XRD图谱与BiOBr-Cu基本一致，表明引入聚吡咯异质界面印迹层并未明显改变BiOBr-Cu的晶体结构。

**图5:**
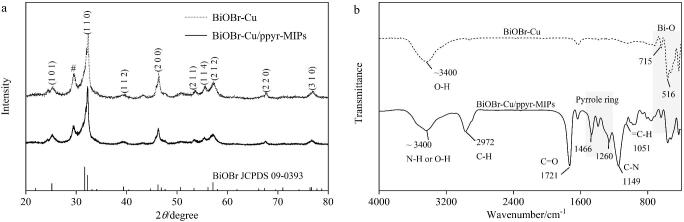
BiOBr-Cu和BiOBr-Cu/ppyr-MIPs的（a）XRD图和（b）FTIR图

#### 2.2.3 FTIR表征

通过FTIR对BiOBr-Cu和BiOBr-Cu/ppyr-MIPs的官能团进行表征，如图5b所示。BiOBr-Cu和BiOBr-Cu/ppyr-MIPs谱图中，均在715 cm^-1^和516 cm^-1^处出现了特征峰，这可以归因于四方相BiOBr中的Bi-O伸缩振动。在BiOBr-Cu样品中，约3 400 cm^-1^处的宽吸收带可归因于自由水分子中O-H的拉伸和弯曲振动。在BiOBr-Cu/ppyr-MIPs样品中，3 400 cm^-1^处的特征峰可以归因于O-H或N-H的拉伸和弯曲振动，在2 972 cm^-1^处出现了归属于C-H的伸缩振动峰，而1 721 cm^-1^处的特征峰可以归因为酯类中C=O的振动。此外，在BiOBr-Cu/ppyr-MIPs谱图中，1 500~1 050 cm^-1^范围内出现了一系列特征峰（1 051、1 149、1 260和1 466 cm^-1^），分别归属于吡咯环上的C-H平面振动、C-H拉伸振动和CN杂环的典型拉伸振动。这些特征峰的出现表明BiOBr-Cu/ppyr-MIPs的成功制备。

#### 2.2.4 XPS表征

通过XPS对BiOBr-Cu和BiOBr-Cu/ppyr-MIPs的表面化学成分和氧化态进行表征。如图6a可知，BiOBr-Cu的Bi 4*f*光谱中在165.27 eV和160.07 eV处出现了两个主特征峰，分别归属于Bi 4*f*_5/2_和Bi 4*f*_7/2_，对应于Bi^3+［21］^。在BiOBr-Cu/ppyr-MIPs的Bi 4*f*谱中，这两个主特征峰向低结合能方向移动，分别出现在164.49 eV和159.23 eV。此外，BiOBr-Cu的Bi 4*f*谱图中还出现了两个小峰，归属于BiOBr-Cu表面出现的空穴（Bi-OVs）^［22］^；而在BiOBr-Cu/ppyr-MIPs谱中这两个峰消失。在Br 3*d*高分辨谱中也观测到了与Bi相似的结合能移动。如图6b所示，BiOBr-Cu的Br 3*d*光谱图中出现两个主特征峰，分别位于70.20 eV和69.10 eV，对应于Br 3*d*_3/2_和Br 4*f*_5/2_^［23］^。在BiOBr-Cu/ppyr-MIPs中，这两个主特征峰向低结合能方向移动，分别位于69.30 eV和68.25 eV。此外，BiOBr-Cu的Br 3*d*谱图中在67.70 eV处出现了一个小峰，归属于游离的Br^-^，而在BiOBr-Cu/ppyr-MIPs谱中未观测到该峰。这些实验结果表明，聚吡咯印迹层与BiOBr-Cu间存在化学相互作用，而非简单的物理混合。

**图6:**
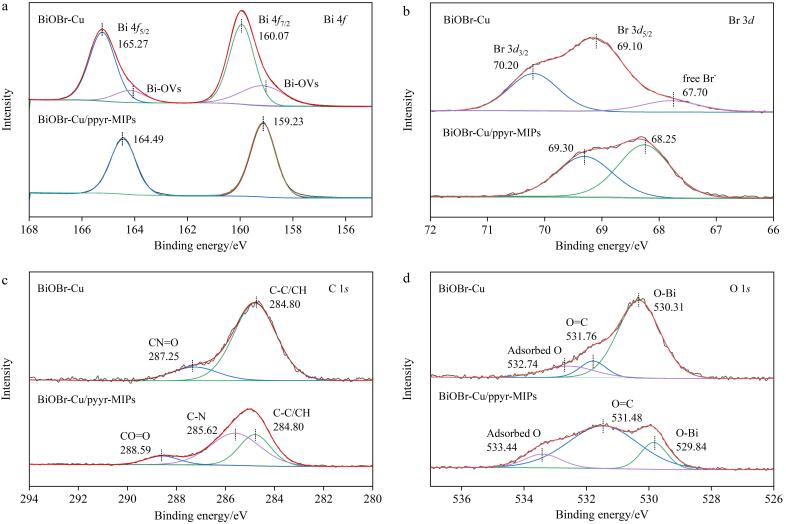
BiOBr-Cu和BiOBr-Cu/ppyr-MIPs的（a）Bi 4*f*、（b）Br 3*d*、（c）C 1*s*和（d）O 1*s*高分辨XPS谱图

图6c中，BiOBr-Cu的C 1*s*高分辨图出现了两个特征峰，位于287.25 eV和284.80 eV处，分别归因于CN=O和C-C/CH两个碳环境。BiOBr-Cu中的C元素可能主要来自XPS测量过程中扩散泵的油污染碳。BiOBr-Cu/ppyr-MIPs的C 1*s*高分辨图出现了288.59、285.62和284.80 eV 3个特征峰，分别归属于O-C=O、C-N和C-C/CH 3种碳环境^［24］^。图6d为O 1*s*的高分辨图，BiOBr-Cu和BiOBr-Cu/ppyr-MIPs谱图均出现3个特征峰，分别归属于吸附O、O=C和O-Bi^［22］^。相较于BiOBr-Cu， BiOBr-Cu/ppyr-MIPs的C 1*s*和O 1*s*谱中归属于C-N和O=C峰的比例均显著增加，进一步证明了BiOBr-Cu/ppyr-MIPs材料的成功制备。

#### 2.2.5 光电性能表征

通过UV-vis DRS对BiOBr-Cu和BiOBr-Cu/ppyr-MIPs的光吸收性能进行表征。如图7a所示，制备得到的BiOBr-Cu在465 nm处出现陡峭的吸收边缘，表明其具有可见光吸收性能。从吸收边缘延伸至550 nm处，出现了一个宽且弱的吸收，这可能是来自于表面的空位及其他缺陷。在550 nm和800 nm之间出现了一个较大的吸收带，归因于掺杂Cu引入的次能级^［25］^。相较于BiOBr-Cu， BiOBr-Cu/ppyr-MIPs在可见光（400~800 nm）范围内的光吸收能力明显增强，表明引入聚吡咯层能够有效拓宽材料的光吸收范围，增强材料对可见光的利用率，从而增强光催化活性^［26］^。

**图7:**
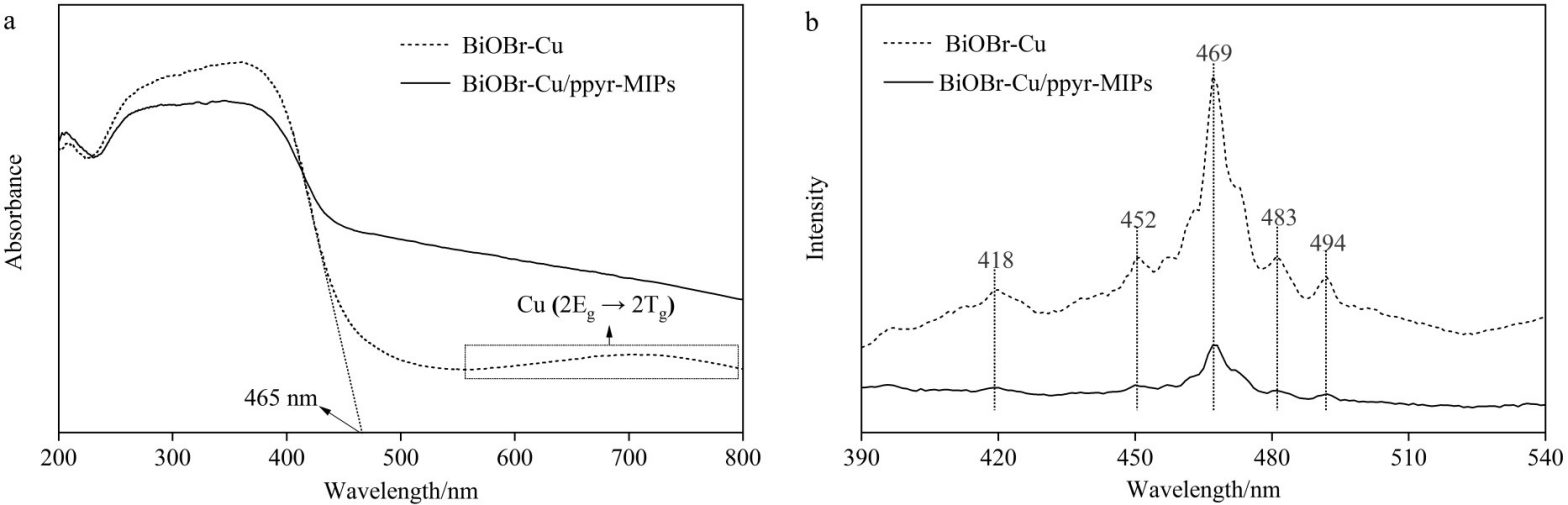
BiOBr-Cu和BiOBr-Cu/ppyr-MIPs的（a）UV-vis DRS图和（b）PL图

通过PL对BiOBr-Cu和BiOBr-Cu/ppyr-MIPs的电荷复合行为进行表征，如图7b所示。在BiOBr-Cu样品中，418 nm处出现了一个发射峰，对应于BiOBr的本征带隙发射。其他出现的发射峰（452、469、483和494 nm）分属于不同的发光中心，如铋间隙、氧空位和铜掺杂引起的缺陷能级^［25］^。通常，PL强度与光生电子-空穴的复合率成正比^［27］^。相较于BiOBr-Cu， BiOBr-Cu/ppyr-MIPs的PL强度显著降低。这表明聚吡咯异质界面印迹层具有良好的光电活性，能够有效抑制光生电子-空穴的复合，从而增强光催化活性^［28］^。

### 2.3 吸附性能研究

BiOBr-Cu/ppyr-MIPs和BiOBr-Cu/ppyr-NIPs的动力学吸附实验如图8a所示。BiOBr-Cu/ppyr-MIPs和BiOBr-Cu/ppyr-NIPs的吸附量在前10 min迅速增加，且BiOBr-Cu/ppyr-MIPs的吸附量高于BiOBr-Cu/ppyr-NIPs，表明聚吡咯印迹层中存在大量与模板分子AO高度匹配的印迹位点。随着印迹位点逐渐被占据，在30 min内BiOBr-Cu/ppyr-MIPs逐渐达到吸附动态平衡。采用伪一级和伪二级动力学模型对BiOBr-Cu/ppyr-MIPs和BiOBr-Cu/ppyr-NIPs的动力学吸附数据进行拟合。图8b为伪二级动力学模型拟合线，从结果可以看出，伪二级动力学拟合相关系数（*R* ^2^）均大于0.990，表明BiOBr-Cu/ppyr-MIPs和BiOBr-Cu/ppyr-NIPs的吸附更符合伪二级动力学模型，在吸附过程中化学吸附为主要限速步骤^［29，30］^。此外，BiOBr-Cu/ppyr-MIPs的吸附速率（44.8 mg/（g·min））是BiOBr-Cu/ppyr-NIPs吸附速率（9.10 mg/（g·min））的4.92倍，这可能是由于BiOBr-Cu/ppyr-MIPs表面存在大量、可及的印迹位点，从而实现对模板分子的快速、选择性吸附。

**图8:**
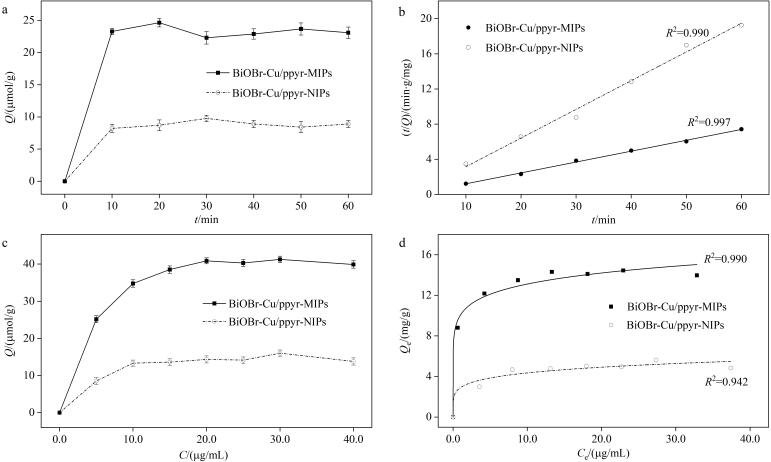
BiOBr-Cu/ppyr-MIPs和BiOBr-Cu/ppyr-NIPs的（a）动力学吸附图、（b）拟二级动力学拟合曲线、（c）等温吸附图以及（d）热力学Freundlich拟合曲线（*n*=3）

BiOBr-Cu/ppyr-MIPs和BiOBr-Cu/ppyr-NIPs的等温吸附实验如图8c所示。随着AO初始浓度增加，BiOBr-Cu/ppyr-MIPs和BiOBr-Cu/ppyr-NIPs的吸附量逐步增加，当质量浓度为20.0 μg/mL时，材料达到吸附平衡。BiOBr-Cu/ppyr-MIPs对AO的平衡吸附量（40.9 μmol/g）是BiOBr-Cu/ppyr-NIPs（13.8 μmol/g）的2.96倍，这可以归因于聚吡咯印迹层内存在高密度印迹位点。采用Freundlich和Langmuir吸附模型对BiOBr-Cu/ppyr-MIPs和BiOBr-Cu/ppyr-NIPs的等温吸附数据进行拟合。图8d为基于Freundlich等温吸附模型的拟合线，从结果可以看出，Freundlich等温吸附模型的*R* ^2^大于Langmuir吸附模型，表明材料吸附更符合Freundlich等温吸附模型，BiOBr-Cu/ppyr-MIPs对AO的吸附主要基于多层吸附机制^［31，32］^。

### 2.4 降解性能研究

#### 2.4.1 影响因素研究

探究了BiOBr-Cu/ppyr-MIPs用量（5.0、10.0、15.0、20.0 mg）对BiOBr-Cu/ppyr-MIPs光降解性能的影响。如图9a和9b所示，当材料用量为5.0 mg时，仅有63.7%的AO被降解，光降解性能较弱。降解率随着材料用量的增加而增加，当材料用量为15.0 mg时，降解率最大，达96.0%，足量的BiOBr-Cu/ppyr-MIPs提供充足的活性位点用于AO的高效降解。然而随着BiOBr-Cu/ppyr-MIPs用量进一步增加至20.0 mg，光降解过程受到抑制，降解率下降至90.7%。这可能是因为过量的BiOBr-Cu/ppyr-MIPs会造成溶液浊度和材料自身团聚可能性增加，导致光利用率和活性位点利用率双双降低，进而影响BiOBr-Cu/ppyr-MIPs的降解效率^［33］^。

**图9:**
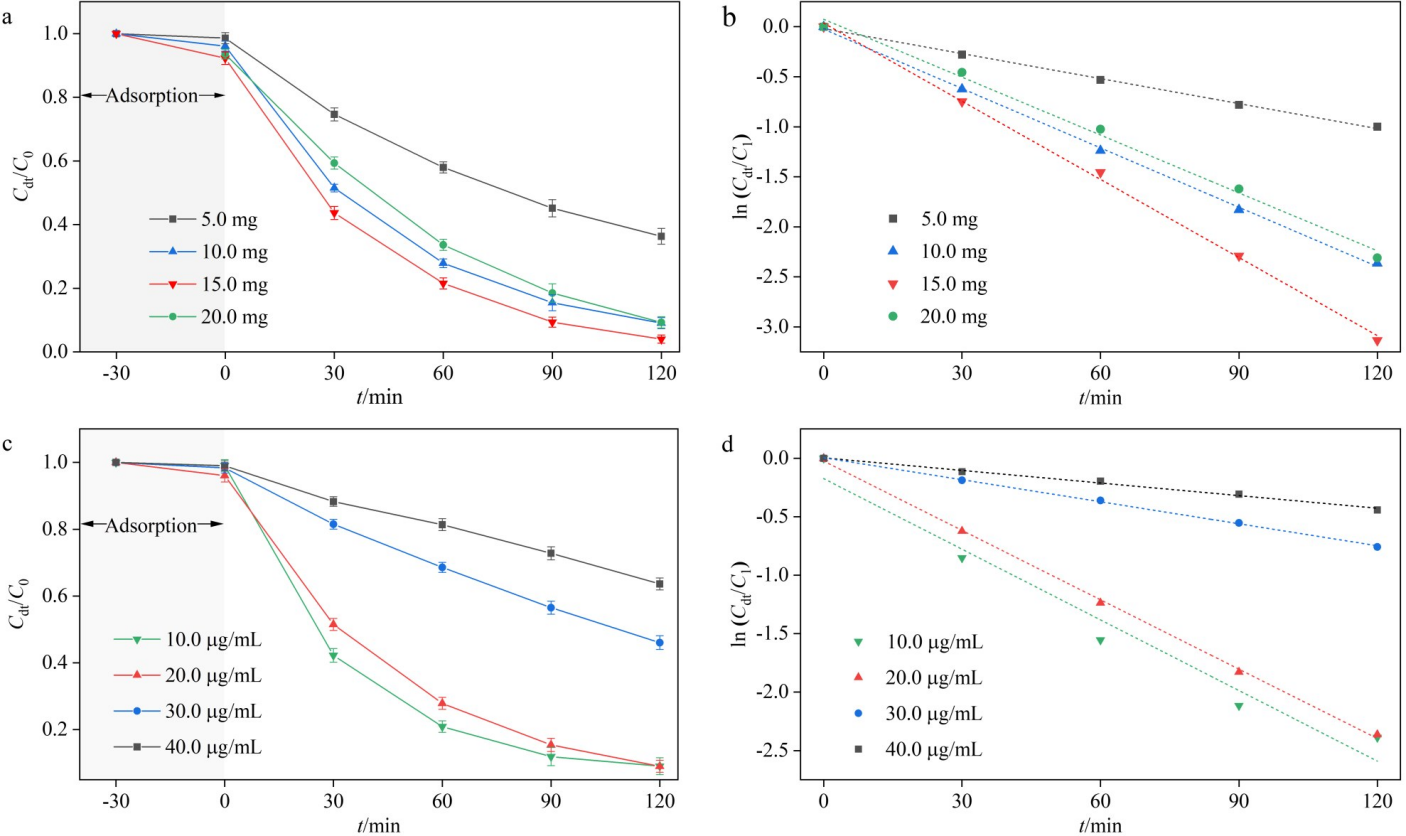
（a）BiOBr-Cu/ppyr-MIPs用量对光降解性能的影响及（b）一级动力学拟合；（c）AO初始质量浓度对光降解性能的影响及（d）一级动力学拟合（*n*=3）

探究了不同污染物初始浓度（10.0、20.0、30.0、40.0 μg/mL）对BiOBr-Cu/ppyr-MIPs光降解性能的影响。如图9c所示，当AO初始质量浓度低于20.0 μg/mL时，BiOBr-Cu/ppyr-MIPs对AO的降解率大于90.9%，在较低的浓度下能够实现高效光降解去除模板分子。动力学拟合显示（图9d），降解速率常数与AO初始浓度呈负相关。这可以从两方面解释：一方面，随着初始浓度的增加，AO分子及降解产生的中间体数量随之增加，导致活性位点竞争加强，从而降低整体降解效率；另一方面，染料浓度的增加会显著提高溶液的色度，导致材料的光子吸收减少，光利用率降低，进而影响BiOBr-Cu/ppyr-MIPs的降解效率^［34，35］^。

探究了不同溶液初始pH值（3、5、7、9、11）对BiOBr-Cu/ppyr-MIPs光降解性能的影响。如图10a和10b所示，当pH值为3~9，AO的降解率保持在75.5%以上。当pH值为5~7时，BiOBr-Cu/ppyr-MIPs展现出优异的降解率和降解效率。这些结果表明BiOBr-Cu/ppyr-MIPs在弱酸性到弱碱性范围内对目标物均具有优异的降解效果。当pH值为11时，AO的降解率显著降低，仅为24.2%。这可能是因为在碱性条件下，AO与BiOBr-Cu/ppyr-MIPs之间存在静电排斥，且溶液中大量存在的氢氧根离子会消耗大量活性物种，从而导致降解率显著降低。

**图10:**
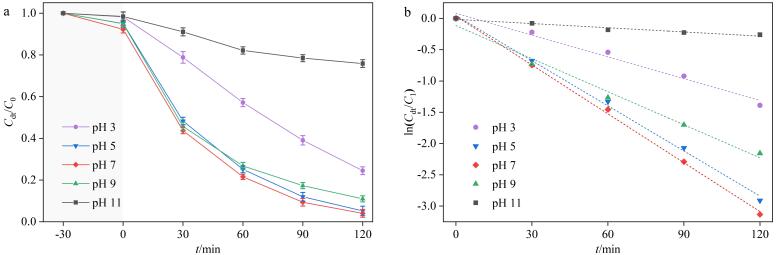
（a）初始pH对光降解性能的影响及（b）一级动力学拟合（*n*=3）

#### 2.4.2 与其他工作对比

为了评估材料的光降解性能，将BiOBr-Cu/ppyr-MIPs与文献中报道的其他铋基光催化复合材料进行比较，结果如表1所示。与其他铋基光催化剂相比较，BiOBr-Cu/ppyr-MIPs在较低的材料用量（15.0 mg，比用量第二少的Bi/Bi_4_NbO_8_Cl少62.5%）、较短时间（120 min）内实现高效降解去除AO（96.0%）。通过计算TOF对光降解性能相关数据进行归一化分析。经计算，BiOBr-Cu/ppyr-MIPs具有最高的TOF，是其他材料2.04~5.79倍。此外，BiOBr-Cu/ppyr-MIPs的TOF是BiOBr-Cu/ppyr-NIPs的2.32倍，表明聚吡咯印迹层中的印迹位点能够协同增强材料的光降解性能。

**表1:** 不同铋基光催化复合材料对AO光降解效率的比较

Materials	Dosage/ mg	Original AO mass concentration/（μg/mL）	Degradation rate/%	Degradation time/min	TOF/ （10^-5^ min^-1^）	Ref.
Ag-Ag_3_O_4_/Bi_4_V_2_O_11_	100	10.0	99.5	180	5.53	［^36^］
Bi/Bi_4_NbO_8_Cl	40.0	20.0	99.9	180	11.1	［^37^］
Ppy/Bi_2_MoO_6_	50.0	50.0	94.3	480	15.7	［^38^］
BiOBr-Cu/ppyr-NIPs	15.0	20.0	41.4	120	13.8	this work
BiOBr-Cu/ppyr-MIPs	15.0	20.0	96.0	120	32.0	this work

TOF： turnover frequency.

#### 2.4.3 重复利用性

在可见光照射下，通过吸附-降解循环实验评估了BiOBr-Cu/ppyr-MIPs的可重复利用性。如图11a所示，经过5个循环后，AO光降解率仍然能够达到初次降解率的90.7%，这表明BiOBr-Cu/ppyr-MIPs具有良好的可重复利用性。降解率的轻微下降可能是由于循环实验中材料质量及活性位点的少量损失。通过FTIR对使用前后的BiOBr-Cu/ppyr-MIPs进行表征。如图11b所示，BiOBr-Cu/ppyr-MIPs的主要官能团结构没有明显变化。这些结果表明，BiOBr-Cu/ppyr-MIPs表现出良好的化学稳定性和可重复使用性。

**图11:**
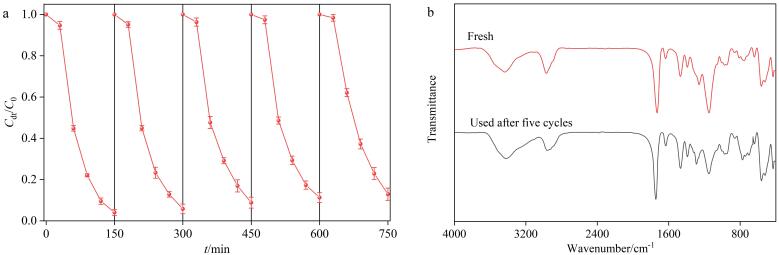
BiOBr-Cu/ppyr-MIPs的重复利用性（*n*=3）

### 2.5 选择性研究

为了评估BiOBr-Cu/ppyr-MIPs的选择性降解性能，选取3种结构类似物（AR、RA、OG）作为AO竞争物。如图12所示，AO和3种结构类似物具有共同的主框架结构，即苯磺酸基偶氮-2-萘酚，仅在取代基上有所不同。BiOBr-Cu/ppyr-MIPs对AO的降解率（96.0%）明显高于其他竞争物。这可能是由于3种结构类似物中额外的取代基引起空间位阻，导致竞争物分子与印迹空穴匹配度不高。其中，相较于RA和OG，BiOBr-Cu/ppyr-MIPs对AR的降解率略高（45.5%），这可能是由于AO和AR取代基数目更相近。此外，BiOBr-Cu/ppyr-NIPs对4种结构类似物降解效果差别不大，降解率较低。这一实验结果表明BiOBr-Cu/ppyr-MIPs对目标分子具有优异的选择性。

**图12:**
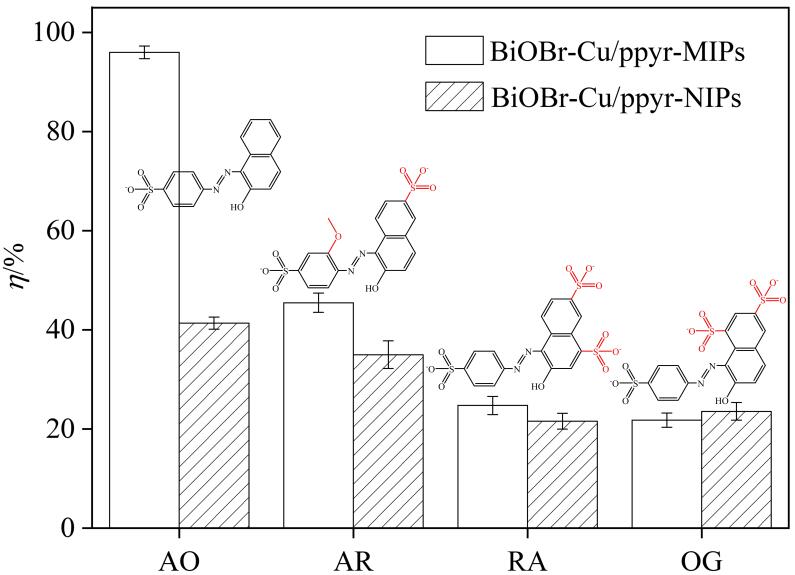
BiOBr-Cu/ppyr-MIPs和BiOBr-Cu/ppyr-NIPs的选择性实验（*n*=3）

为了进一步研究BiOBr-Cu/ppyr-MIPs的选择性，采用选择性系数（*k*_selectivity_）作为指标，评估材料的选择性降解性能。如表2所示，基于BiOBr-Cu/ppyr-MIPs的*k*_imprinted_值≥2.11，且对OD的*k*_imprinted_值可达4.40，表明BiOBr-Cu/ppyr-MIPs对于模板分子具有优异的识别能力。相较之下，基于BiOBr-Cu/ppyr-NIPs的*k*_comparison_值≥1.18，表明非印迹材料对几种污染物分子的降解存在微小的差异，这可能与分子的空间位阻效应以及功能基团的数量有关。对于整个体系来讲，*k*_selectivity_值≥1.79，表明BiOBr-Cu/ppyr-MIPs对AO具有较好的选择性，印迹空穴在选择性降解过程中发挥重要作用。

**表2:** BiOBr-Cu/ppyr-MIPs和BiOBr-Cu/ppyr-NIPs的降解率和选择性系数

Analyte	*η*_MIPs_/%	*η*_NIPs_/%	*k*_imprinted_	*k*_comparsion_	*k*_selectivity_
AR	45.5	35.0	2.11	1.18	1.79
RA	24.8	21.6	3.87	1.92	2.02
OG	21.8	23.6	4.40	1.75	2.51

### 2.6 选择性降解机理研究

#### 2.6.1 活性物种捕获实验

探究了BiOBr-Cu/ppyr-MIPs降解过程的主要活性物种。如图13所示，在加入IPA后，降解率（92.8%）变化不大，表明光降解过程没有受到明显抑制。在加入TEOA和LAA后，AO的降解率分别为37.9%和35.6%，表明光降解过程受到明显的抑制。这些实验结果表明，·O_2_^-^和空穴（h^+^）是BiOBr-Cu/ppyr-MIPs降解去除AO过程中的主要活性物种。

**图13:**
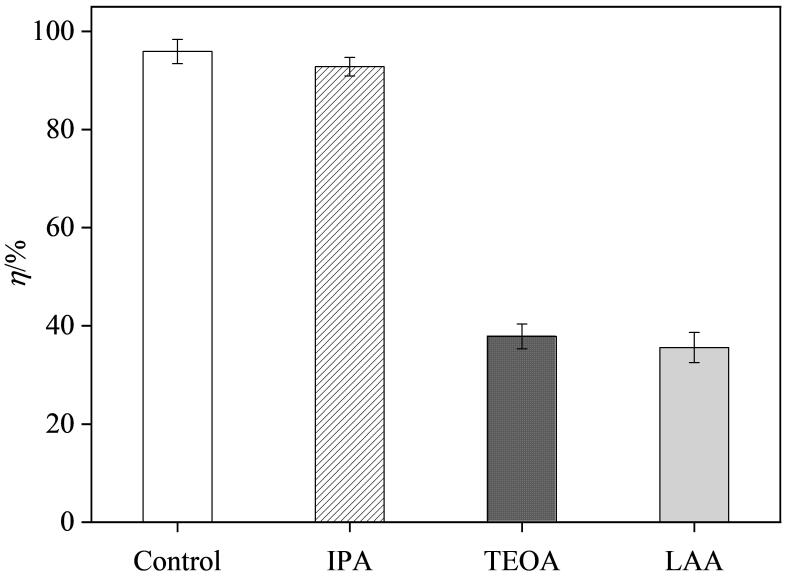
不同捕获剂对BiOBr-Cu/ppyr-MIPs光降解性能的影响（*n*=3）

#### 2.6.2 光催化机理分析

图14a描述了BiOBr-Cu/ppyr-MIPs可能的电荷传输机理。在可见光照射下，聚吡咯层最高占据分子轨道（HOMO）上的电子通过*π-π* ^*^跃迁至最低未占分子轨道（LUMO）上生成光生电子（e^-^），并在HOMO上留下强氧化性的空穴h^+^。同时，位于内层的BiOBr-Cu载体也被光激发，电子从价带（valence band，VB）跃迁至导带（conduction band，CB）上，产生光生电子-空穴对。处于*π* ^*^轨道的激发态电子直接注入BiOBr-Cu的CB上。相对应地，BiOBr-Cu价带上的h^+^则转移至聚吡咯印迹层的HOMO。最终，电子积累在BiOBr-Cu的CB中，空穴则聚集在聚吡咯层的HOMO中，实现光生电子-空穴对的高效转移和分离。

**图14:**
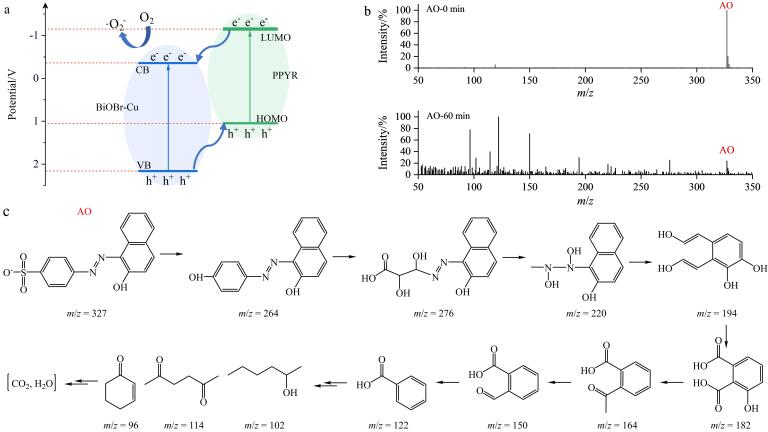
（a）BiOBr-Cu/ppyr-MIPs电荷传输机理图，（b）初始（0 min）和60 min时光降解的质谱图， （c）BiOBr-Cu/ppyr-MIPs光降解AO的可能途径

采用HPLC-MS对降解过程中生成的中间产物进行检测分析。如图14b所示，AO分子的*m*/*z*峰位于327。在降解60 min后，谱图中出现多个新的*m*/*z*信号峰，表明AO分子被有效降解为多个中间产物。基于此，提出AO可能的光降解途径（图14c）。在光照下，BiOBr-Cu价带上的h^+^迁移到聚吡咯层的HOMO中，进攻静电势较负的磺酸基团，使得C-S键断裂，脱去磺酸基形成1-（（4-羟苯基）二氮烯基）萘-2-醇，随后通过氧化、取代和添加，形成萘基化合物和单环芳香族化合物^［39］^。这些中间体被进一步氧化成小分子，如环己烷-2-烯-1-酮、2，5-己二酮和2-己醇。在持续的可见光照射下，小分子最终被矿化成CO_2_和H_2_O等无机化合物。

值得注意的是，文献报道的AO光降解过程通常包含两种主要途径：活性物种攻击偶氮键和活性物种攻击磺酸基^［39-42］^。其中，活性物种攻击偶氮键的降解途径通常会产生*m*/*z*为172的中间体对氨基苯磺酸钠。然而，在本工作中，并未观察到明显的*m*/*z* 172峰。我们可以推测，BiOBr-Cu/ppyr-MIPs在降解AO时，主要经历了活性氧对磺酸基的攻击途径。这可能是由于AO优先吸附在聚吡咯层中的印迹位点，能够与迁移至聚吡咯层的h^+^直接发生氧化反应，表明聚吡咯印迹层在一定程度上能够实现降解路径的选择性。

## 3 结论

本工作通过将表面分子印迹技术引入BiOBr/ppyr复合材料中，成功制备得到高降解效率分子印迹光催化剂BiOBr-Cu/ppyr-MIPs。BiOBr-Cu/ppyr-MIPs在可见光范围内的光吸收显著增强，光生电子-空穴快速分离，转化频率高出其他材料2.04~5.79倍，展现出优异的光降解效率。此外，一系列实验表明所制备的材料具有高选择性、高吸附容量以及高吸附速率。本工作所提出的异质界面原位印迹策略为开发高降解效率分子印迹光催化剂提供了新思路。

## References

[1] Firmansyah M L， Ashraf M， Ullah N . Sep Purif Technol， 2025， 360： 131111

[2] Lee S Y， Tan Y H， Lau S Y， et al . Environ Res， 2024， 259： 119448 38942255 10.1016/j.envres.2024.119448

[3] Saleem M H， Mfarrej M， Khan K A， et al . Sci Total Environ， 2024， 913： 169755 38176566 10.1016/j.scitotenv.2023.169755

[4] Sobiech M， Luliński P . TrAC-Trends Anal Chem， 2024， 174： 117695

[5] Arabi M， Ostovan A， Li J H， et al . Adv Mater， 2021， 33（30）： 2100543 10.1002/adma.202100543 34145950

[6] Shen X T， Zhu L H， Li J， et al . Chem Commun， 2007， 11： 1163 10.1039/b615303h 17347726

[7] Liu X， Zhu L， Wang X， et al . J Nanopart Res， 2020， 22： 300

[8] Du Q Z， Wu P， Sun Y Y， et al . Chem Eng J， 2020， 390： 124614

[9] Bi L B， Chen Z L， Li L H， et al . J Hazard Mater， 2021， 407： 124759 33341571 10.1016/j.jhazmat.2020.124759

[10] Zhang J， Tian X M， Dong C C， et al . New J Chem， 2023， 47（15）： 7278

[11] Zhou G Z， Cao Y Y， Jin Y Q， et al . J Cleaner Prod， 2020， 274： 122929

[12] Li L L， Zheng X Y， Chi Y H， et al . J Hazard Mater， 2020， 383： 121211 31546219 10.1016/j.jhazmat.2019.121211

[13] Wang R， Guo M， Hu Y L， et al . ACS Omega， 2020， 5（32）： 20664 32832820 10.1021/acsomega.0c03095 PMC7439697

[14] Guo M， Hu Y L， Wang R， et al . Environ Res， 2021， 194： 110684 33417912 10.1016/j.envres.2020.110684

[15] Sun Y， Szulejko J E， Kim K H， et al . Chin J Catal， 2023， 55： 20

[16] Tian X M， Wang P C， Wang Y， et al . J Mater Chem A， 2024， 12： 18381

[17] Yang Y C， Wen J W， Wei J H， et al . ACS Appl Mater Interfaces， 2013， 5（13）： 6201 23767991 10.1021/am401167y

[18] Zhu P， Zhang Y， Zhang D W， et al . Polymers， 2023， 15（3）： 538 36771840 10.3390/polym15030538 PMC9920636

[19] Hussain A， Hou J， Tahir M， et al . Catal Rev Sci Eng， 2022， 66（1）： 119

[20] Huo Y， Zhang J， Miao M， et al . Appl Catal， B， 2012， 111/112： 334

[21] Kang S， Pawar R C， Pyo Y， et al . J Exp Nanosci， 2016， 11（4）： 259

[22] Sheng H， Wang W， Dai R， et al . Nanomaterials， 2021， 11（2）： 423 33562318 10.3390/nano11020423 PMC7914912

[23] Guan Z P， Li Q Y， Shen B， et al . Sep Purif Technol， 2020， 234： 116100

[24] Xu J J， Hu Y M， Zeng C， et al . J Colloid Interface Sci， 2017， 505： 719 28662474 10.1016/j.jcis.2017.06.054

[25] Lv X C， Yan D Y S， Lam F L， et al . Chem Eng J， 2020， 401： 126012

[26] Tran V V， Nu T T V， Jung H， et al . Polymers， 2021， 13（18）： 3031 34577932 10.3390/polym13183031 PMC8470106

[27] Mo H B， Chen Q， Wang D， et al . J Mater Chem C Mater， 2024， 12（6）： 2025

[28] Fan Y Z， Wang Z G， Ren W Z， et al . ACS Appl Mater Interfaces， 2022， 14（32）： 36966 35921222 10.1021/acsami.2c11503

[29] He Q， Liang J J， Chen L X， et al . Water Res， 2020， 168： 115164 31629229 10.1016/j.watres.2019.115164

[30] Zhou S， Fu J L， Zhao P F， et al . Sep Sci Plus， 2022， 5（8）： 337

[31] Wang S， Yang J Y， Sun J Q， et al . Food Chem， 2023， 426： 136621 37354582 10.1016/j.foodchem.2023.136621

[32] Stachowiak M， Cegłowski M， Kurczewska J . Int J Biol Macromol， 2023， 251： 126356 37595706 10.1016/j.ijbiomac.2023.126356

[33] Huang Q W， Lou X P， Nie D X， et al . Sep Purif Technol， 2024， 347： 127518

[34] Luo T W， Bao Y H . Sep Purif Technol， 2024， 347： 127503

[35] Yaseen M， Khan A， Humayun M， et al . Green Chem Lett Rev， 2024， 17（1）： 2321251

[36] Esmaeili M， Haghighi M， Shabani M， et al . J Environ Chem Eng， 2024， 12（1）： 111634

[37] Wu X L， Zhang Y L， Wang K， et al . J Hazard Mater， 2020， 393： 122408 32143158 10.1016/j.jhazmat.2020.122408

[38] Sun H P， Liu Z， Liu X Q， et al . Mater Today Sustainability， 2022， 19： 100154

[39] Feng S， Xiao B， Wu M， et al . Sep Purif Technol， 2020， 248： 117004

[40] Shen J H， Horng J J， Wang Y S， et al . Chemosphere， 2017， 182： 364 28505578 10.1016/j.chemosphere.2017.05.043

[41] Wang X Y， Xu G Z， Tu Y Z， et al . Chem Eng J， 2021， 411： 128456

[42] Le T X H， Nguyen T V， Yacouba Z A， et al . Chemosphere， 2016， 161： 308 27441990 10.1016/j.chemosphere.2016.06.108

